# Increased expression of the yeast multidrug resistance ABC transporter Pdr18 leads to increased ethanol tolerance and ethanol production in high gravity alcoholic fermentation

**DOI:** 10.1186/1475-2859-11-98

**Published:** 2012-07-27

**Authors:** Miguel C Teixeira, Cláudia P Godinho, Tânia R Cabrito, Nuno P Mira, Isabel Sá-Correia

**Affiliations:** 1 IBB – Institute for Biotechnology and BioEngineering, Centro de Engenharia Biológica e Química, and Department of Bioengineering, Instituto Superior Técnico, Technical University of Lisbon, Av. Rovisco Pais, 1049-001, Lisbon, Portugal

**Keywords:** *Saccharomyces cerevisiae*, Ethanol tolerance, ABC multidrug transporters, Membrane permeabilization, Bio-ethanol production

## Abstract

**Background:**

The understanding of the molecular basis of yeast tolerance to ethanol may guide the design of rational strategies to increase process performance in industrial alcoholic fermentations. A set of 21 genes encoding multidrug transporters from the ATP-Binding Cassette (ABC) Superfamily and Major Facilitator Superfamily (MFS) in *S. cerevisiae* were scrutinized for a role in ethanol stress resistance.

**Results:**

A yeast multidrug resistance ABC transporter encoded by the *PDR18* gene, proposed to play a role in the incorporation of ergosterol in the yeast plasma membrane, was found to confer resistance to growth inhibitory concentrations of ethanol. *PDR18* expression was seen to contribute to decreased ^3^ H-ethanol intracellular concentrations and decreased plasma membrane permeabilization of yeast cells challenged with inhibitory ethanol concentrations. Given the increased tolerance to ethanol of cells expressing *PDR18*, the final concentration of ethanol produced during high gravity alcoholic fermentation by yeast cells devoid of *PDR18* was lower than the final ethanol concentration produced by the corresponding parental strain. Moreover, an engineered yeast strain in which the *PDR18* promoter was replaced in the genome by the stronger *PDR5* promoter, leading to increased *PDR18* mRNA levels during alcoholic fermentation, was able to attain a 6 % higher ethanol concentration and a 17 % higher ethanol production yield than the parental strain. The improved fermentative performance of yeast cells over-expressing *PDR18* was found to correlate with their increased ethanol tolerance and ability to restrain plasma membrane permeabilization induced throughout high gravity fermentation.

**Conclusions:**

*PDR18* gene over-expression increases yeast ethanol tolerance and fermentation performance leading to the production of highly inhibitory concentrations of ethanol. *PDR18* overexpression in industrial yeast strains appears to be a promising approach to improve alcoholic fermentation performance for sustainable bio-ethanol production.

## Background

*Saccharomyces cerevisiae* is extensively used in wine-making and brewing processes, and in bio-ethanol production [1]. The successful performance of these industrial alcoholic fermentations depends on the ability of the used yeast strains to cope with a number of stress factors occurring during the process [2-4]. Stress induced by increasing amounts of ethanol, accumulated to highly inhibitory toxic concentrations during yeast alcoholic fermentation, is the major responsible for reduced ethanol productivity and for stuck and sluggish fermentations [5]. Thus, yeast strains that can endure stress imposed by high ethanol concentrations are highly desired.

A number of studies based on detailed physiological and molecular analyses [2,6-8] or on genome-wide surveys [9-15] have contributed to increase the understanding of the processes underlying ethanol toxicity and yeast tolerance to stress induced by this metabolite. Among the determinants of ethanol stress resistance identified so far, *FPS1*, described as an aquaglyceroporin involved in the control of the intracellular level of glycerol [16-21], has been reported to influence the intracellular accumulation of ethanol, upon sudden exposure to this compound [15]. Although the exact mechanism of Fps1 action in this context is not clear, Fps1 may affect ethanol partitioning either directly [15] or through its effect in plasma membrane ergosterol content [22]. Consistently, increased *FPS1* expression was shown to increase the final ethanol concentration achieved by yeast cells, in conditions leading to high ethanol titters [15].

In the present study, the participation of a number of plasma membrane multidrug resistance (MDR) transporters of the ATP-Binding Cassette (ABC) superfamily (Pdr5, Pdr10, Pdr11, Pdr12, Pdr15, Pdr18, Snq2 and Yor1) and Major Facilitator Superfamily (MFS) (Aqr1, Atr1, Azr1, Dtr1, Flr1, Qdr1, Qdr2, Qdr3, Tpo1, Tpo2, Tpo3 and Tpo4) in ethanol stress resistance was scrutinized. Being implicated in the resistance to a variety of chemical stresses and involved in yeast detoxification from these compounds [23,24], multidrug resistance transporters are plausible candidates for conferring yeast resistance to ethanol. Based on this screening, *PDR18*, encoding a plasma membrane [25] multidrug resistance transporter of the ABC superfamily [26], was the sole MDR transporter found to confer resistance to toxic concentrations of ethanol. Pdr18 was recently characterized as conferring resistance to chemical stress agents, including the herbicides 2,4-dichlorophenoxyacetic acid (2,4-D) and barban, the agricultural fungicide mancozeb, and to cadmium, copper, manganese and zinc [26]. Based on a genome-wide screening, *PDR18* expression was also found to confer resistance to the anticancer drugs cisplatin and carboplatin and the antifungal drug nocodazole [27]. Pdr18 was found to play a role in plasma membrane sterol incorporation, and this physiological trait proposed to contribute to its action as a multidrug resistance determinant [26]. Indeed, the demonstrated effect that *PDR18* expression has in plasma membrane ergosterol concentration and transmembrane potential is likely to affect transport across cell membranes and drug partition between the cell interior and the extracellular medium [26]. The participation of Pdr18 in reducing the intracellular concentration of ethanol in yeast cells was examined in this study and *PDR18* expression found to increase yeast ability to achieve higher final concentrations of ethanol during alcoholic fermentation. An engineered *S. cerevisiae* strain, in which the natural *PDR18* promoter was replaced by the stronger *PDR5* promoter, was obtained and shown to attain higher ethanol concentrations in a high gravity fermentation-like medium.

## Results

### The expression of the ABC transporter Pdr18 increases yeast tolerance to ethanol

**Figure 1:**
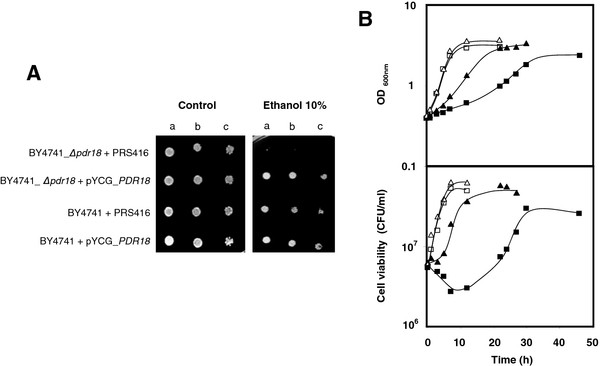
***PDR18*expression increases yeast tolerance to ethanol.** Comparison of the susceptibility to ethanol-induced stress of *S. cerevisiae* parental strain BY4741 and the derived deletion mutant *Δpdr18*, through spot assays (**A**) or cultivation in liquid medium (**B**). Cells used for the ethanol susceptibility assays were mid-exponential phase cells (OD_600nm_ = 0.5±0.05) cultivated in minimal growth medium in the absence of stress. **A**: The susceptibility to inhibitory concentrations of ethanol of BY4741 and derived *Δpdr18* strains, harboring a *PDR18* expression plasmid or the corresponding cloning vector pRS416 was compared. The cell suspensions used to prepare the spots in lanes **b**) and **c**) were 1:5 and 1:25 serial dilutions, respectively, of the suspensions with an OD_600nm_ = 0.05±0.005 in lane **a**). **B**: Wild-type (▵, ▴) and *Δpdr18* (□, ■) cell suspensions were also used to inoculate MM4 liquid medium in the absence (▵, □) or presence (▴, ■) of 6 % ethanol, with an initial OD_600nm_ = 0.05±0.005. Growth curves, followed by the measurement of the OD_600nm_ and the number of colony forming units (CFU) per ml, are representative of at least three independent experiments.

The susceptibility towards ethanol-induced stress (6-12 % (v/v)) of 21 single deletion mutants, each devoid of a multidrug resistance transporter of the ABC superfamily - *Δpdr5*, *Δpdr10*, *Δpdr11*, *Δpdr12*, *Δpdr15*, *Δsnq2*, *Δyor1* or *Δpdr18* – or of the MFS - *Δaqr1*, *Δatr1*, *Δazr1*, *Δdtr1*, *Δflr1*, *Δqdr1*, *Δqdr2*, *Δqdr3*, *Δtpo1*, *Δtpo2*, *Δtpo3*, *Δtpo4* or *Δyhk8* -, was compared to the parental strain, BY4741, susceptibility based on spot assays. Among the tested strains, only mutant *Δpdr18*, with the *PDR18* gene deleted, exhibited decreased susceptibility towards ethanol stress (Figure 1A). The deletion of *PDR18* was found to lead to a more extended lag-phase period (10 h compared to 2 h for the parental strain) when the yeast cell population was cultivated in liquid medium supplemented with 6 % ethanol (Figure 1B). This lag-phase was found to correspond to an initial period of viability loss for strain *Δpdr18* culture, after which the surviving population was able to resume exponential growth in the presence of ethanol, although exhibiting a decreased maximum specific growth rate, when compared to the parental strain culture, whose growth was also inhibited (Figure 1B). The expression of *PDR18* from a centromeric plasmid was found to rescue the ethanol susceptibility phenotype registered in *Δpdr18* cells, to levels comparable to the parental strain, while no effect is seen in the presence of the corresponding cloning vector (Figure 1A).

### *PDR18* expression decreases intracellular ethanol accumulation and ethanol-induced plasma membrane permeabilization in yeast

**Figure 2:**
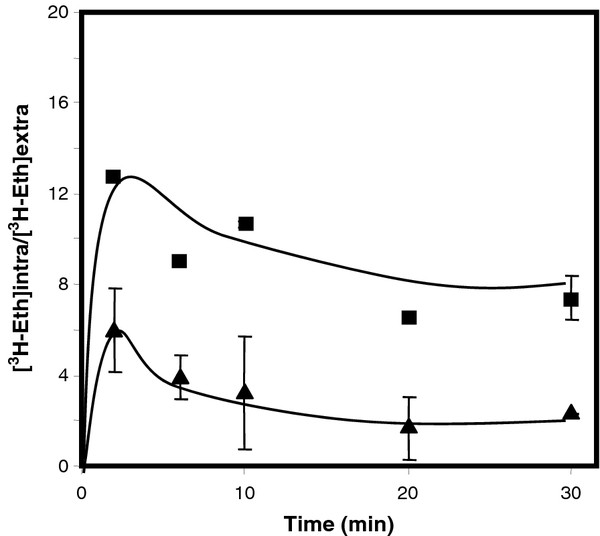
***PDR18*expression decreases intracellular ethanol accumulation.** Comparison of [^3^ H]-ethanol accumulation in non-adapted cells of *S. cerevisiae* parental strain BY4741 (▴) and the derived deletion mutant *Δpdr18* (■), during cultivation for 30 min in MM4 liquid medium supplemented with 6 % cold ethanol. The accumulation ([^3^ H-Eth]intra/[^3^ H-Eth]extra) values are the means of at least three independent experiments, error bars indicating standard deviation.

Given the role of Pdr18 as a multidrug resistance transporter involved in ergosterol incorporation in the yeast plasma membrane [26] and the results reported herein showing that it confers ethanol tolerance in yeast, the effect of *PDR18* expression in the intracellular accumulation of ^3^ H-ethanol was assessed. Wild-type and *Δpdr18* yeast cells were cultivated in the absence of stress till mid-exponential growth phase and transferred to media containing a moderately inhibitory concentration of ethanol (6 % (v/v)) (Figure 2), mimicking the conditions used in the start of the growth experiment shown in Figure 1B. The accumulation of ^3^ H-ethanol in yeast cells was registered during the first 30 min of cultivation under these conditions. The intracellular/extracellular accumulation ratio of ^3^ H-ethanol was found to be around 2-fold higher in cells devoid of the *PDR18* gene, indicating that Pdr18 activity contributes to decreased ethanol accumulation in yeast cells.

**Figure 3:**
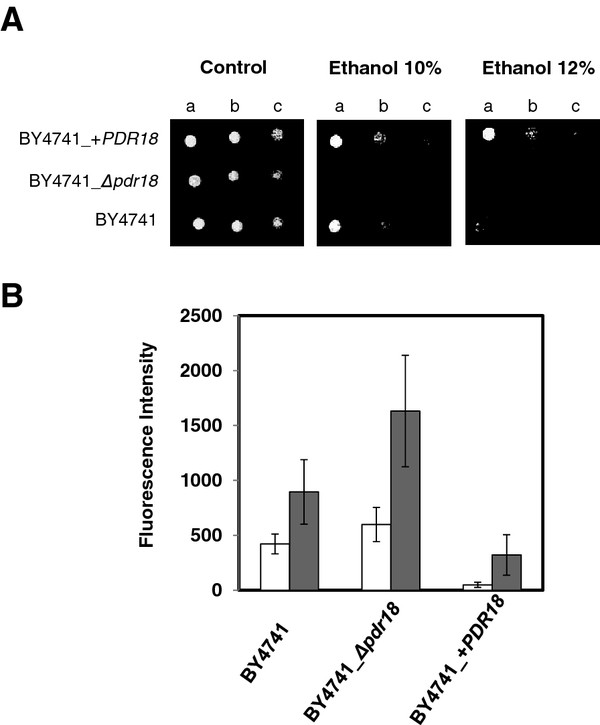
***PDR18*over-expression increases ethanol tolerance and protects from ethanol-induced plasma membrane permeabilization.A**: Comparison of the susceptibility to ethanol induced stress of *S. cerevisiae* parental strain BY4741 and the derived deletion mutant BY4741_*Δpdr18*, and *PDR18* over-expression strain BY4741_ + *PDR18* through spot assays. Cells used for the spot assays were mid-exponential phase cells (OD_600nm_ = 0.5±0.05) cultivated in minimal growth medium in the absence of stress. The cell suspensions used to prepare the spots in lanes **b**) and **c**) were 1:5 and 1:25 serial dilutions, respectively, of the suspensions with an OD_600nm_ = 0.05±0.005 in lane a). **B**: Comparison of the membrane permeability exhibited by *S. cerevisiae* yeast strains BY4741, BY4741_*Δpdr18* and BY4741_ + *PDR18*, grown to mid-exponential phase and exposed (white columns) or not (grey columns) for 30’ to mild-stress induced by 4 % ethanol. Cell permeability was assessed based on the fluorescence intensity exhibited by yeast cells upon passive accumulation of propidium iodide. Fluorescence intensity values are the means of at least three independent experiments, error bars indicating standard deviation.

To assess whether an increased expression of *PDR18* in the parental strain could further increase its level of ethanol tolerance and ethanol production, the natural *PDR18* promoter region, in its chromosomal location, was replaced by the stronger *PDR5* promoter region in the BY4741 strain, as described in the Methods section. The choice of the *PDR5* promoter was based on its relatively high strength and on its sustained transcriptional up-regulation registered during alcoholic fermentation [28]. The choice for the manipulation of *PDR18* expression from its chromosomal locus, instead of introducing extra copies of the gene in expression plasmids, was based on the fact that it allows the use of this strain in high gravity rich medium, similar to those used for industrial bio-ethanol production, in which the selective pressure required for plasmid maintenance cannot be assured. This engineered yeast strain, over-expressing the *PDR18* gene, was found to exhibit an increased tolerance towards very high ethanol concentrations, allowing cell growth at ethanol concentrations close to those lethal for the wild-type strain (Figure 3A).

Given the Pdr18 role in ergosterol incorporation in the plasma membrane, the effect of Pdr18 expression on ethanol-induced plasma membrane permeabilization was examined. The measurement of the intracellular accumulation of the fluorescent marker propidium iodide was used as an indirect method to assess plasma membrane permeability. Based on this method, the permeability of ethanol-stressed yeast cells was seen to depend deeply on the expression of *PDR18* (Figure 3B). Indeed, even in control conditions *Δpdr18* deletion mutant cells exhibited a nearly 2-fold increase in fluorescence levels, compared to the parental strain, whereas the *PDR18* over-expressing strain displayed a 10-fold decrease in cell fluorescence. Even a mild concentration of ethanol (4 %) was found to be sufficient to increase membrane permeability. In these stress conditions, the expression of *PDR18* was found to be required to restrain ethanol-induced plasma membrane permeabilization (Figure 3B).

### High gravity alcoholic fermentation with an engineered yeast strain exhibiting increased *PDR18* expression levels produces a higher final ethanol concentration

**Figure 4:**
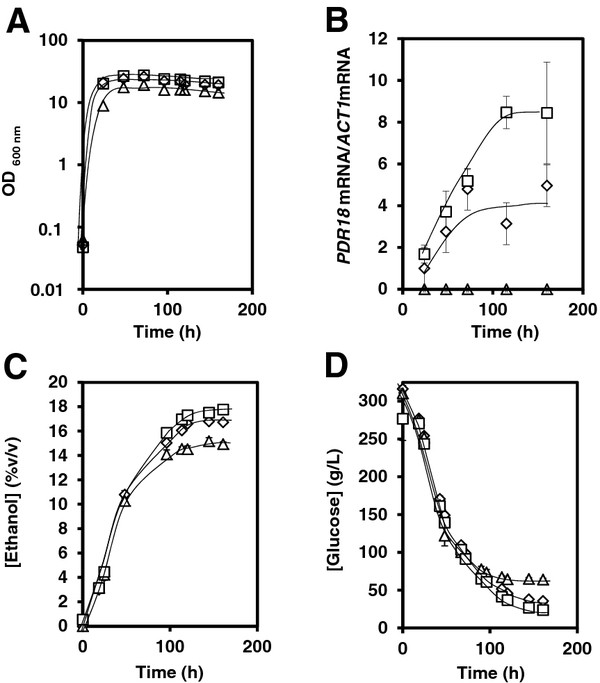
***PDR18*over-expression leads to higher ethanol production yield in a high gravity-like fermentation medium.** Comparison of the *PDR18* transcript levels (**B**), and the extracellular concentrations of ethanol (**C**) and glucose (**D**), accumulated/used during cultivation in fermentation medium, containing 30 % glucose, of *S. cerevisiae* yeast strains BY4741 (◊), BY4741_*Δpdr18* (▵) and BY4741_ + *PDR18* (□). **A**: Growth curves, followed by measuring culture OD_600nm_, are representative of at least three independent experiments. **B**: The relative values of *PDR18* mRNA/*ACT1* mRNA, as obtained through RT-PCR, are the means of at least three independent experiments, error bars indicating standard deviation. The *PDR18* mRNA relative value for the parental BY4741 in control conditions was set as 1 and the remaining values were calculated relative to it. **C** and **D**: Ethanol and glucose concentration levels, assessed using HPLC, are also mean values of at least three independent experiments, error bars indicating standard deviation.

To assess whether or not the role of Pdr18 in plasma membrane ergosterol composition and ethanol homeostasis could have an impact in the performance of alcoholic fermentation, BY4741 cells, and the derived *Δpdr18* and *PDR18*-overexpression strains, were cultivated in liquid YPD medium, containing 30 % (w/v) glucose and additional supply of amino acids for which the strain is auxotrophic. Despite being a laboratory medium, this medium is usually accepted as a good system to study high gravity fermentation conditions [29] leading to high ethanol production (up to 17 % (v/v)), most of which is produced by yeast cells in stationary-phase caused by the limitation of some nutrient other then glucose (Figure 4A). Interestingly, the *PDR18* transcript levels, measured through RT-PCR, were found to be up-regulated progressively throughout the fermentation progression (Figure 4B). This up-regulation was observed in both the wild-type strain and the *PDR18* over-expression strain, the latter exhibiting in all collected time-points an approximately 2-fold higher *PDR18* expression level (Figure 4B). Yeast cells devoid of the *PDR18* gene were found to be unable to reach the same level of final ethanol production as the wild-type population, exhibiting a reduction of 10 % in the ethanol concentration reached upon 160 h of fermentation (Figure 4C). When comparing the parental strain and the *Δpdr18* deletion mutant, glucose consumption appears to be proportional to the levels of ethanol produced (Figure 4D). Indeed, the ethanol production yield was found to be similar for both strains - around 47.5 g ethanol produced/g glucose consumed. The engineered yeast strain over-expressing *PDR18*, on the other hand, was able to exhibit a more than 6 % increase in the final concentration of ethanol attained after 160 h, when compared to the parental strain (Figure 4C). Furthermore, this engineered strain was found to exhibit a 17 % higher ethanol production yield, reaching 55.6 g ethanol produced/g glucose consumed, when compared to that of the parental strain. It is interesting to observe that until around 14 % (v/v) ethanol is reached, ethanol production rate in cells expressing or not the *PDR18* gene is very similar. However, once higher ethanol concentrations are attained, stationary phase cells expressing different levels of *PDR18* exhibit differential ability to proceed with alcoholic fermentation (Figure 4C). *Δpdr18* cells arrest alcoholic fermentation when 15 % (v/v) ethanol is reached, while wild-type cells continue fermentative metabolism up to nearly 17 % (v/v) ethanol and the *PDR18* over-expression strain was able to produce a final concentration of around 18 % (v/v) ethanol (Figure 4C).

**Figure 5:**
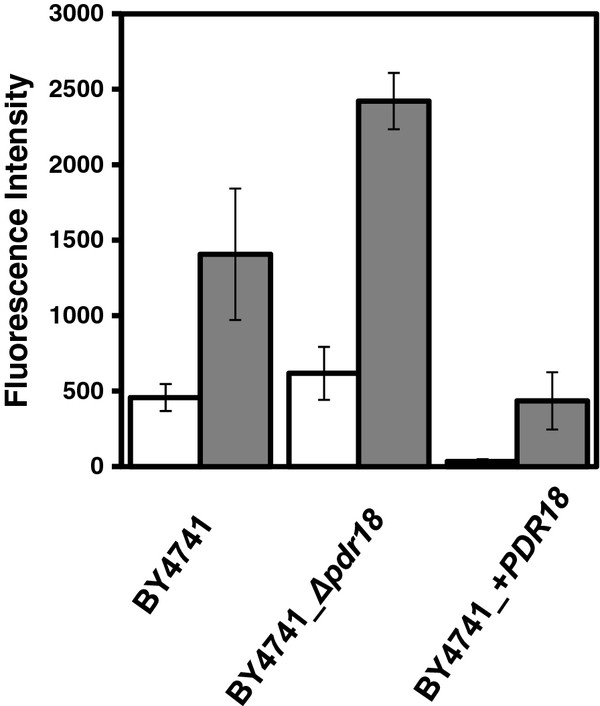
***PDR18*over-expression protects from fermentation-induced plasma membrane permeabilization.** Comparison of the membrane permeability exhibited by *S. cerevisiae* yeast strains BY4741, BY4741_*Δpdr18* and BY4741_ + *PDR18*, upon 6 h (white columns; corresponding to exponentially growing cells) or 160 h (grey columns; corresponding to stationary phase cells, upon fermentation arrest) of cultivation in fermentation medium containing 30 % glucose. Cell permeability was assessed based on the fluorescence intensity exhibited by yeast cells upon passive accumulation of propidium iodide. Fluorescence intensity values are the means of at least three independent experiments, error bars indicating standard deviation.

The observation that Pdr18 plays a role in counteracting plasma membrane permeabilization in ethanol stressed-cells led us to check for a similar role during fermentation leading to high ethanol concentrations. Upon 6 h of cultivation in fermentation medium, containing an initial glucose concentration of 30 %, when yeast cells are already growing exponentially but still experiencing a high-glucose concentration of around 25 %, a dramatic effect of Pdr18 expression in membrane permeability was observed. While the *Δpdr18* deletion mutant cells exhibited higher fluorescence intensity, when compared to the parental BY4741 strain, the *PDR18* over-expression cells displayed a 13-fold decrease in plasma membrane permeability (Figure 5). After 160 h of cultivation, when fermentation is already arrested, yeast plasma membrane permeability is increased in all strains (Figure 5), consistent with the accumulation of toxic concentrations of ethanol and other toxic fermentation side-products. Under these conditions, Pdr18 expression appears again to contribute to decrease stress-induced plasma membrane permeabilization (Figure 5), which correlates with the ability of the *PDR18* over-expressing strain to exhibit an improved fermentative performance.

## Discussion

This study describes the protective role of the yeast ABC transporter Pdr18 against ethanol stress, and the subsequent development of an engineered yeast strain able to achieve higher final ethanol concentrations in a high gravity fermentation media suitable for bio-ethanol production.

Based on a screening of mutants devoid of MFS or ABC multidrug transporters, *PDR18* was found to be a determinant of resistance to inhibitory concentrations of ethanol in *Saccharomyces cerevisiae*. *PDR18* gene expression was shown to decrease the intracellular accumulation of radiolabelled ethanol against a concentration gradient in stressed yeast cells. Although it is surprising to find out that a lipophilic molecule such as ethanol can be accumulated in yeast cells, a previous report had shown that ethanol also accumulates intracellularly in the absence of the Fps1 aquaglyceroporin [15], or during the first hours of alcoholic fermentation in media containing 20 % glucose [30]. Whether Pdr18 facilitates the direct efflux of ethanol across the yeast plasma membrane, or indirectly affects its intracellular accumulation, remains to be established. Given that apparently a highly lipophylic molecule such as ethanol does not require a transporter since it may easily cross the lipid bilayer by passive diffusion, it is likely that its accumulation in yeast cells may occur at the level of the plasma membrane and depend on its lipid composition. This is a possible explanation for the differential accumulation of ethanol in yeast cells devoid of *FPS1*[15] or *PDR18* (this work), whose deletion is known to affect at least the plasma membrane ergosterol composition [22,26].

In this work it was found that Pdr18 expression contributes to increased ethanol tolerance, by controlling ethanol-induced plasma membrane permeabilization. The results obtained herein, correlating the decrease in *PDR18* expression with the increase in plasma membrane permeability, are consistent with the effect of Pdr18 in the incorporation of ergosterol in the yeast plasma membrane [26]. Indeed, the sterol plasma membrane composition has been shown to affect the level of plasma membrane permeabilization under stress conditions, particularly those leading to dehydration and changes in cell volume, a described effect of ethanol stress [31].

The obtained results led us to hypothesize that Pdr18 expression could also increase yeast capability to produce higher ethanol concentrations, being an advantage for industrial processes. Indeed, in conditions that lead to high ethanol production (medium containing 30 % glucose and no other limiting nutrient), the deletion of *PDR18* was shown to reduce the concentration of ethanol reached when compared to the wild-type strain, whereas the engineered *PDR18*-overexpressing strain was shown to reach even higher ethanol concentrations in this industrial-like fermentation medium. Furthermore, the *PDR18*-overexpressing yeast strain was found to exhibit an increased ethanol production yield, favouring an improved use of the available glucose. This observation correlates with the effect of Pdr18 in restraining plasma membrane permeabilization occurring throughout alcoholic fermentation. It may also be related to the role of Pdr18 in ergosterol incorporation in the yeast plasma membrane, since a recent study shows that increasing ergosterol concentration in model membranes protects the membrane by preventing the ethanol-induced formation of an interdigitated phase, maintaining optimal membrane thickness as ethanol concentration increases during anaerobic fermentations [32]. The over-expression of *PDR18* in industrial yeast strains is pointed out as a promising strategy to further increase bio-ethanol production yield in industrial-scale processes. The strategy used in this study might also be a good choice for the manipulation of *PDR18* expression in such industrial strains, since the *PDR5* promoter is stronger than the natural *PDR18* promoter and was shown in this study to lead to higher transcriptional levels during alcoholic fermentation than that induced by the *PDR18* promoter. This choice further allows the use of high gravity rich media, similar to those used for industrial bio-ethanol production, in which the selective pressure required for plasmid maintenance cannot be assured or would become too expensive.

## Conclusions

Higher ethanol yield is highly desired to reduce production costs associated to bio-ethanol production, in order to increase the sustainability of this process of obtaining energy from renewable sugar-rich substrates. In this study, *PDR18* expression was found to increase yeast tolerance to highly inhibitory concentrations of ethanol, possibly through its role in decreasing ethanol-induced plasma membrane permeabilization and reducing the intracellular ethanol concentration. The final ethanol production reached by yeast cells in fermentation medium leading to high ethanol production was found to be higher in yeast cells over-expressing this gene. The over-expression of the *PDR18* gene from its chromosomal locus in industrial yeast strains appears to be a promising strategy to increase the capacity for sustainable production of higher ethanol concentrations in industrial processes.

## Methods

### Strains, plasmids and growth media

The parental *Saccharomyces cerevisiae* strain BY4741 (*MATa, his3Δ1, leu2Δ0, met15Δ0, ura3Δ0*) and the derived single deletion mutants *Δpdr5*, *Δpdr10*, *Δpdr11*, *Δpdr12*, *Δpdr15*, *Δpdr18*, *Δsnq2*, *Δyor1*, *Δaqr1*, *Δatr1*, *Δazr1*, *Δdtr1*, *Δflr1*, *Δqdr1*, *Δqdr2*, *Δqdr3*, *Δtpo1*, *Δtpo2*, *Δtpo3*, *Δtpo4*, *Δyhr048w* and *Δyor152c* (all obtained from the EUROSCARF collection) were used in this study. A plasmid pRS416_*PDR18*, expressing the *PDR18* gene from its natural promoter, and the corresponding cloning vector, pRS416 (both plasmids were obtained from the EUROSCARF collection), were also used for complementation assays. Cells were cultivated in either rich YPD medium, containing 20 g/l glucose (Merck), 10 g/l Yeast Extract (Difco) and 20 g/l Peptone (Difco), or in minimal medium MM4, containing (per liter): 1.7 g yeast nitrogen base without amino acids or NH4^+^ (Difco), 20 g glucose (Merck), 2.65 g (NH4)_2_SO4 (Merck), 20 mg methionine (Sigma), 20 mg histidine (Sigma), 60 mg leucine (Sigma) and 20 mg uracil (Sigma). Strains were preserved in YPD medium, supplemented with 30 % glycerol, and stored at −80 °C.

### PDR18 over-expression strain construction

To obtain the *PDR18* over-expressing strain BY4741_ *+ PDR18*, the natural *PDR18* promoter (considered to start around -1000 bp from the start codon) was replaced in *S. cerevisiae* BY4741 for the stronger promoter of the *PDR5* gene through homologous recombination. The replacement cassette was obtained by PCR amplification of the *PDR5* promoter region preceded by a kanamycin cassette. This cassette was amplified from the *Δyor152c* deletion mutant, in which the *YOR152c* ORF, located at 1500 bp upstream of the *PDR5* start codon, was replaced by a kanamycin cassette. DNA amplification was carried out using total DNA extracted from *Δyor152c* strain as a template and the following primers: 5’-ATGTCAGCGTGCCGCATTGAAAGGTAAAAACTAAAATTAATGAACTTTTC*GGCGATTTTTGTGTTTCGTC*-3’ and 5’-CTCAATTCCGTATCGCCGTCTTTAACAGTATGGAAATCCATTATGTTTAG*GTCGAGTGATAACTAACACAG*-3’. These primers include 50 nucleotides homologous to the PDR18 promoter region at the 5’ end and 20 and 21 nucleotides homologous to the flanking region of *PDR5* promoter in the *Δyor152c* mutant (in italic), respectively. The PCR product of around 3000 bp was purified and used to transform *S. cerevisiae* BY4741. Transformants in which homologous recombination took place were selected on YPD supplemented with geneticin (150 mg/L) and promoter replacement was confirmed by PCR.

### Ethanol susceptibility assays

The susceptibility of the parental strain BY4741 and derived mutant strains to toxic concentrations of ethanol was assessed by comparing their growth curves or growth on spot assays in MM4 medium supplemented or not with inhibitory concentrations of ethanol (6 % in liquid medium and 10-12 % in solid medium). Cell suspensions used to prepare the inocula for the growth curves or the spot assays were grown in MM4 medium until mid-exponential phase (OD_600nm_ = 0.5±0.05). Cell growth in liquid media was conducted in 100 ml Erlenmeyer flasks, containing 50 ml of growth medium, at 30 °C, 250 rpm, and was followed by measuring culture OD_600nm_ during batch cultivation. Cell suspension samples were diluted to an OD_600nm_ below 0.4±0.05, prior to OD_600nm_ determination. Cell suspensions used for the spot assays were diluted in sterile water to obtain suspensions with OD 600 nm = 0.05 ± 0.005. These cell suspensions and subsequent dilutions (1:5; 1:25) were applied as 4 μl spots onto the surface of agarized MM4 medium, supplemented with adequate ethanol concentrations. In the assays using wild-type or *Δpdr18* cells harbouring the *PDR18* expression plasmid of the corresponding empty vector, cells were grown in MM4-uracil selective medium.

### [^3^ H]-ethanol accumulation assays

Accumulation of ^3^ H-ethanol was assessed as described before [15]. To estimate the accumulation of ethanol (Intracellular/Extracellular ^3^ H-ethanol) from yeast cells, the parental strain BY4741 and the mutant strain *Δpdr18* were grown in MM4 medium till mid-exponential phase. Cells were washed and resuspended in MM4 medium, to obtain dense cell suspensions (OD600nm = 5.0 ± 0.2, equivalent to approximately 2.2 mg (dry weight) ml-1). After 5 minutes incubation at 30 °C, with agitation (150 rev/min), to thermostat the suspensions, 0.1 μM of ^3^ H-ethanol (American Radiolabelled Chemicals; 250 μCi/ml) and 6 % (v/v) of unlabelled ethanol were added to the cell suspensions. Incubation proceeded for an additional period of 30 min. During this period of incubation, the intracellular accumulation of labeled ethanol was followed by filtering 200 μl of cell suspension, at adequate time intervals, through pre-wetted glass microfibre filters (Whatman GF/C). The filters were washed with ice-cold TM buffer (0.1 M MES (Sigma), 41 mM Tris (Sigma) adjusted to pH 5.5 with HCl) and the radioactivity measured in a Beckman LS 5000TD scintillation counter. Extracellular ^3^ H-ethanol was estimated, by radioactivity assessment of 50 μl of the supernatant. Non-specific ^3^ H-ethanol adsorption to the filters and to the cells (less than 5 % of the total radioactivity) was assessed and taken into consideration. To calculate the intracellular concentration of labeled ethanol, the internal cell volume (Vi) of the exponential cells, grown in the absence of drug and used for accumulation assays, was considered constant and equal to 2.5 μl (mg dry weight)-1 [7].

### Membrane permeability measurement assays

Cell membrane permeability for populations of BY4741, BY4741_*Δpdr18* and BY4741_ *+ PDR18* strains was compared by fluorescence microscopy, using as a fluorescent marker propidium iodide (PI), whose accumulation is considered to be dependent on membrane permeability [33]. Cells were harvested by centrifugation (8,600 x *g* for 5 minutes at 4 °C) at suitable time points during the fermentation process and after 30 minutes of growth in the absence or presence of ethanol (4 %). Cell pellets were ressuspended in 500 mL fresh medium to an OD_600nm_ = 0,4. PI (30 μM, 750 μL; Sigma) was added to the cell suspensions and incubated in the dark with orbital agitation (15 minutes, 250 rpm). PI-exposed cells were harvested by centrifugation (17,500 x g for 5 minutes) and washed twice and ressuspended in distilled deionized water. Fluorescence was examined with an Axioplan microscope equipped with adequate epifluorescence interface filters (BP450-490 and LP520; Zeiss). Fluorescence emission was collected with a cooled charge-coupled device camera (Cool SNAPFX; Roper Scientific Photometrics), and the images were analyzed with MetaMorph, version 3.5. The fluorescence images were background corrected by using dark-current images. Only the fluorescence of living cells was considered, unviable cells being identified based on whole-cell dispersion of fluorescence emitted upon incorporation of DAPI (4',6-diamidino-2-phenylindole; 50 ng/ml) staining and bright-field analysis of cell morphology. The fluorescence intensity values, considered to be proportional to PI accumulation, were calculated as the average of the fluorescence intensity of a minimum of 50 cells/experiment. The value of fluorescence intensity emitted by each cell was calculated by the software as the average of pixel by pixel intensity in the selected region of interest.

#### Assessment of ethanol and glucose concentration during alcoholic fermentation

The parental strain BY4741, the derived deletion mutant *Δpdr18* and the *PDR18* overexpressing strain BY4741_ *+ PDR18* were grown in YPD medium, supplemented with glucose (Merck), in order to obtain a final concentration of 300 g/l, and with the amino acids for which the strain is auxotrophic (240 mg/L Leucine, 80 mg/L Histidine and 80 mg/L Methionine). Cell suspensions used to prepare the inocula were grown in YPD medium until mid-exponential phase (OD_600nm_ = 0.5±0.05). Cell growth in liquid media was followed by measuring culture OD_600nm_ during batch cultivation. Samples of culture supernatants were harvested by centrifugation and used for the quantification of ethanol and glucose concentrations by HPLC. Cultures supernatants were analysed on an Aminex HPX-87 H Ion Exchange Chromatography column, eluted at room temperature with 0.005 M H_2_SO_4_ at a flow-rate of 0.6 mL min^-1^ during 30 minutes, using a refractive-index detector. Under such experimental conditions glucose had a retention time of 8.3 minutes and ethanol 19.4 minutes. Reproducibility and linearity of the method were tested and concentrations were estimated based on appropriate calibration curves.

## Competing interests

The authors declare that they have no competing interests.

## Authors’ contributions

MCT carried out phenotypic and transport assays. CG and TRC carried out phenotypic and gene expression analysis and the plasma membrane permeability assessment. CG and NPM conducted the fermentation assays. MCT and ISC designed and coordinated the study and wrote the manuscript. All authors read and approved the final manuscript.
